# Unveiling the roadblocks: exploring substance use disorder treatment policies in Iran through a qualitative lens

**DOI:** 10.1186/s13722-024-00511-4

**Published:** 2024-11-12

**Authors:** Saeid Mirzaei, Vahid Yazdi-Feyzabadi, Mohammad Hossein Mehrolhassani, Nouzar Nakhaee, Nadia Oroomiei

**Affiliations:** 1 Non Communicable Diseases Research Center, Bam University of Medical Sciences, Sardaran Shahid Square-Shahid Rajaei Boulevard, Bam, 7616913555 Iran; 2 Health Services Management Research Center, Institute for Futures Studies in Health, Kerman University of Medical Sciences, Kerman, Iran; 3 Medical Informatics Research Center, Institute for Futures Studies in Health, Kerman University of Medical Sciences, Kerman, Iran; 4 Health Services Management Research Center, Institute for Futures Studies in Health, Kerman University of Medical Sciences, Kerman, Iran

**Keywords:** Policy analysis, Treatment, Harm reduction, Qualitative study, Substance abuse, War on drugs, Moral model, Disease model, Iran

## Abstract

**Background:**

Different countries, including Iran, have implemented various policies to address substance use disorder. This study aims to describe the policies related to substance use disorder treatment and identify challenges related to these policies in Iran since the beginning of the Iranian Revolution in 1979.

**Methods:**

This qualitative study utilized document analysis and interviews with policymakers and implementers. We reviewed a total of 22 documents related to substance use disorder treatment and harm reduction. The results from document analysis complemented and validated the interview data. The research population comprised policymakers and implementers, including individuals directly involved in formulating and implementing substance use disorder treatment policies. Purposive sampling was employed, with a snowball strategy utilized to maximize diversity. Data saturation was achieved after conducting 32 semi-structured interviews. Conventional content analysis was used for data analysis.

**Results:**

In general, the policy landscape for substance use disorder treatment in the Islamic Republic of Iran can be divided into two periods: the “Moral Model” era (1979–1993) and the “Disease Model” era (1993–present). Challenges within the content of substance use disorder treatment policies in Iran encompass the lack of law revisions, existence of contradictions in laws and nature of disease, the absence of evidence-based policymaking, and an inadequate comprehensive perspective on the phenomenon of substance use disorder.

**Conclusions:**

The presence of multiple authorities with different perspectives on substance use disorder and its treatment, coupled with the application of personal preferences in policymaking and the absence of evidence-based policymaking, have contributed to weaknesses in decision-making and policy formulation. The true philosophy of Disease Model appears not to have been fully grasped by health policymakers in Iran, as all Disease Model policies have been pursued with an emphasis on abstinence and quitting. Iran and other nations facing similar challenges should place more reliance on evidence-based approaches and shift away from the “Moral Model” paradigm to develop more effective substance use disorder treatment policies.

## Background

Substance-related disorders and their negative consequences are considered a significant global public health issue today [1]. Substance use disorder has witnessed a substantial increase in recent years, leading to a humanitarian crisis, with cannabis being the most commonly used drug worldwide at 228 million users, followed by opioids (60 million), amphetamines (30 million), cocaine (23 million), and ecstasy (20 million) [2].

Various approaches have been employed to combat this problem for a long time. Supply reduction was the preferred strategy for addressing substance-related issues in many countries for years [3]. This approach gained widespread acceptance globally but incurred significant costs [4].

Over time, more studies have been conducted to understand the reasons for substance use disorder worldwide. The findings of these studies indicate that substance use disorder is a multifaceted and complex phenomenon resulting from the interaction of various factors. Consequently, addressing this issue solely through the supply reduction strategy, despite continuous law enforcement and supply reduction efforts, has become less prominent. The focus has shifted to the demand reduction domain, which includes various interventions for treating substance use disorders [5]. These interventions have gained widespread acceptance and have experienced significant global expansion [4], including in Iran.

Iran, a religious Islamic country in the Middle East and North Africa (MENA) region [6], holds a strategically sensitive position due to its geographic proximity to Afghanistan and Pakistan, sharing approximately 1800 km of borders. Afghanistan has become the world’s largest producer of opium and heroin, serving as the shortest transit route for drugs from neighboring countries in the east to the west. Around 80% of the world’s heroin originates in Afghanistan, with more than three-quarters of these substances trafficked through Iran and Pakistan [7]. Thus, Iran’s strategic location poses a double threat to Iranian society [8, 9].

Global statistics indicate that the number of people with alcohol or drug use disorders in Iran increased from 434,987 cases in 1990 to 955,756 in 2021 [10]. Additionally, the mortality rate attributed to substance use disorders per 100,000 population in Iran rose from 2.621 in 2000 to 2.689 in 2021 [11]. Statistics from 2018 indicate that there are 437,000 substance injectors in the MENA region, with approximately 200,000 of them residing in Iran [12].

Similar to other countries, Iran has long pursued a war on drugs policy. However, in 2004, the Welfare Organization initiated a shift towards harm reduction policies. Over the years, Iran has implemented various treatment policies addressing substance use disorders [13]. While several studies on substance use disorder have been conducted in Iran [14–16], there has been limited emphasis on the policy aspect of this issue. Therefore, the present study aims to describe the policies related to substance use disorder treatment and identify challenges related to these policies in Iran since the beginning of the Iranian Revolution in 1979.

## Methods

This study aimed to describe the policies related to substance use disorder treatment and identify challenges related to these policies in Iran through a combination of document analysis and interviews with policymakers and implementers. In this context, the term ‘documents’ encompasses all relevant publications, policies, laws, and protocols related to substance use disorder treatment and harm reduction in Iran. These documents span from January 1979 to October 2023, have been publicly released, and are accessible.

The research team identified key high-level documents by conducting library searches and reviewing the websites of various entities, including the Expediency Discernment Council, Drug Control Headquarters, General Office for Treatment and Social Support of the Drug Control Headquarters, Ministry of Health, Treatment, and Medical Education, Welfare Organization, Islamic Consultative Assembly (Parliament), and the National Social Council. To ensure that the most important documents in the field were reviewed, the research team employed an approach. This included, in addition to searching websites, consulting participants in the study as experts to recommend relevant documents. Additionally, the research team searched for keywords such as ‘demand side,’ ‘drug use/treatment,’ and ‘harm reduction’ to find relevant documents. Inclusion criteria for this phase included documents that refer to substance use treatment and harm reduction (focusing on the demand side), as well as documents foundational to policymaking in this field. Exclusion criteria included documents focusing on the supply side or those for which the full text is not accessible. Finally, the selection of relevant documents followed Jupp’s criteria, encompassing aspects such as authenticity, credibility, representativeness, and meaning [17].

In total, 22 documents underwent review (Table 1). According to the research objectives, these documents were reviewed, extracted and analyzed separately by three members of the research team. The findings derived from the document analysis were utilized to complement and validate the data obtained from interviews.

The research participants consisted of policymakers and implementers involved in the substance use disorder treatment policy process in Iran. This encompassed individuals with significant knowledge and roles in entities such as the Ministry of Health, Treatment, and Medical Education, Welfare Organization, Drug Control Headquarters, Prison Organization, and National Expediency Discernment Council at the national, provincial, and local levels. The research sample included well-informed key interviewees from these entities and their affiliated organizations, who directly contributed to the formulation and implementation of substance use disorder treatment policies. The sampling approach employed a purposive strategy, utilizing snowball and maximum diversity techniques. Selection criteria involved a minimum of three years of experience in substance use disorder treatment policy and implementation, familiarity with the policymaking and implementation processes, expertise in the field, and willingness to participate in the research. Data collection during this phase involved semi-structured interviews guided by an interview protocol. Research questions include: What have been the treatment policies of the Islamic Republic of Iran in addressing substance use from the beginning of the Islamic Revolution (1979) to the present? From which year have these policies been implemented? Were these policies implemented simultaneously, or were they completely separate? In your opinion, what challenges are associated with these policies? Data saturation was achieved after conducting 32 interviews. The characteristics and profiles of the interviewees are summarized in Tables 2 and 3.

### Data analysis process

The analysis of the interview data was conducted using a systematic and inductive coding approach. Researchers transcribed the recorded information verbatim onto paper as soon as possible after each interview and listened to the recordings multiple times. Simultaneous note-taking and data recording occurred throughout the research process.

Initially, the research team performed primary coding of the interview transcripts, identifying key concepts and recurrent themes using MAXQDA software, version 2020. The primary codes included ‘old laws’ and ‘policy making based on personal opinions.’ These primary codes were then categorized into broader themes through an iterative process, involving grouping related codes into categories based on their content and significance. For instance, codes related to ‘old regulations,’ ‘outdated regulations,’ or ‘regulations that are not up-to-date’ were grouped under the theme ‘Lack of Revision in Laws,’ while codes related to ‘anecdotal policymaking’ or ‘policy making based on personal opinions’ were categorized under ‘Lack of Evidence-Based Policy-Making.’

In addition to analyzing interview data, the research team reviewed relevant documents to supplement the findings. This involved a comprehensive examination of high-level documents, which were analyzed for insights into policies that have been developed and their challenges in this field. The document reviews were conducted using a similar coding approach, ensuring consistency across data sources. The findings from the document analysis were integrated with the interview data to provide a more complete understanding of the issues at hand.

To ensure the reliability of the coding process, multiple researchers independently coded a subset of transcripts and documents, then compared results to resolve discrepancies. Points of disagreement were reviewed and summarized in meetings with the research team members. The final categories and themes were refined through discussions among the team to ensure they accurately reflected the data. Conventional content analysis was used for the overall data analysis to achieve a comprehensive understanding of the text from both documents and interviews.

### Data validity and reliability

To ensure credibility, the interviews conducted and the extracted content by the research team were referred back to the interviewees, and their feedback was incorporated. Additionally, both interview and document analysis methods were employed to enhance the credibility of the findings. Moreover, for dependability, two members of the research team were involved in content extraction from the interviews. Any discrepancies were discussed and resolved in team meetings. Confirmability and transferability were achieved through the preservation of research documents throughout the qualitative research process and researchers’ prolonged engagement with the research data. To enhance transferability, the entire research process was thoroughly documented to facilitate future replication, and external checks were recorded. It is important to note that this manuscript is part of a larger research project, which resulted in the publication of a separate article [18] focusing on a different aspect of the study.

**Table 1:** Reviewed documents

Name of the documents
Constitution of the Islamic Republic of Iran [19]
First Development Plan of Economic, Social, and Cultural Affairs of the Islamic Republic of Iran [20]
Second Development Plan of Economic, Social, and Cultural Affairs of the Islamic Republic of Iran [21]
Third Development Plan of Economic, Social, and Cultural Affairs of the Islamic Republic of Iran [22]
Fourth Development Plan of Economic, Social, and Cultural Affairs of the Islamic Republic of Iran [23]
Fifth Development Plan of Economic, Social, and Cultural Affairs of the Islamic Republic of Iran [24]
Sixth Development Plan of Economic, Social, and Cultural Affairs of the Islamic Republic of Iran [25]
Vision of the Islamic Republic of Iran in the Horizon of 2025 [26]
Law on the Comprehensive Welfare and Social Security System Structure [27]
Health System Transformation Plan of the Islamic Republic of Iran [28]
Legislative Bill on Intensifying Punishment for Drug-Related Crimes and Security and Treatment Measures for the Rehabilitation and Employment of Addicts [29]
General Policies of the Regime for Combating Drugs [30]
Guideline for establishment, management and supervision of authorized, Non-Governmental, Private or NGO centers for the treatment and harm reduction of drug misuse [31]
Law on the Amendment of the Anti-Narcotics Law and the Addition of Articles to It, Ratified by the Expediency Council in 1997 (with amendments and additions in 2010) [32]
Guidelines on Policies and Approaches for the Treatment of Stimulant Abuse and Dependence in Iran [33]
Collection of Executive Regulations and Guidelines for the Establishment, Management, and Supervision of Authorized Treatment and Harm Reduction Centers for Addicts under Article 15 of the Anti-Narcotics Law Amendment of 1997 (Ratified in August 2010 by the Expediency Council) [34]
Comprehensive Document on Social Support and Addiction Treatment in the Country [35]
Executive Regulations on Custody, Treatment, and Harm Reduction Centers for Addicts under Article 16 of the Anti-Narcotics Law, with Subsequent Amendments and Additions [36]
National Division of Labor Plan for Controlling and Reducing Social Harms [37]
Guidelines for Supervision, Evaluation, and Handling of Violations in Authorized Treatment and Harm Reduction Centers [38]
Regulations on Post-Release Care for Addicts [39]
General policies of the sixth development plan of the Islamic Republic of Iran [40]

**Table 2:** Distribution of interviewees at different levels

Level	Executive positions	Number	Professional experience (years)
National	Head of the Office of Mental Health, Social Health, and Addiction, Ministry of Health, Treatment, and Medical Education	1	3
	Experts from the Office of Mental Health, Social Health, and Addiction, Ministry of Health, Treatment, and Medical Education	2	6 3
	Expert from the Prevention and Treatment Department of drug abuse, Office of Mental Health, Social Health, and Addiction, Ministry of Health, Treatment, and Medical Education	1	3
	Member of the Treatment Committee, Drug Control Headquarters	2	4 3
	Member of the Independent Committee on drug abuse, Expediency Discernment Council	2	8 5
	Deputy for Addiction Prevention, Welfare Organization of Iran	2	3 3
Provincial	Experts from the Addiction Office, Welfare Organization, Kerman Province	2	4 6
	Deputy for Addiction Prevention, Welfare Organization, Kerman Province	1	3
	Specialists in Addiction at Kerman University of Medical Sciences	2	14 7
	Experts from the Department of Mental Health, Social Health, and Addiction, Kerman University of Medical Sciences	2	6 4
	Monitoring and Accreditation Unit, Kerman University of Medical Sciences	2	3 5
	Substance use disorder Specialist, Monitoring and Accreditation Unit, Kerman University of Medical Sciences	2	4 5
	Expert from the Office of Mental Health, Social Health, and Addiction	1	4
	Secretariat of the Coordination Council for drug abuse, Kerman	1	3
	Therapist of the Coordination Council for drug abuse, Kerman	1	5
	Experts from the Coordination Council for drug abuse, Kerman	2	4 7
	Health and Treatment Department of Kerman Central Prison	2	6 3
Local	Responsible personnel in Methadone Maintenance Treatment Centers, Therapeutic Community, Article 16 Centers/ Drug courts, and Drop-in center in Kerman City	4	5 3 4 7
	Total	32	–

**Table 3:** Characteristics of interviewees

Characteristic	*N* (%)
*Age (years)*	
35–45	19 (60%)
45–55	10 (31%)
Older than 55	3 (9%)
*Gender*	
Female	10 (31%)
Male	22 (69%)
*Educational level*	
Bachelor’s	6 (19%)
Master’s	2 (6%)
Ph.D./Medical Doctor	24 (75%)

### Ethical considerations

It should be noted that the present study has received ethical approval from Bam University of Medical Sciences (IR.MUBAM.REC.1402.093). Before conducting interviews, informed consent was obtained from all participants, including their agreement to participate in the research, audio recording, and data implementation. It should be mentioned that this manuscript is part of a broader research project titled “Setting the policy agenda for the treatment of substance use disorders in Iran [18]”.

### Findings

The study’s findings are divided into two parts: content analysis of policies and the identification of content challenges. In the section on content challenges, all quotes are attributed to a specific interviewee using the notation [I], followed by the interviewee’s number. For example, interviewee number 1 is referenced as [I.1].

Substance use disorder treatment policies in Iran from the beginning of the revolution until now fall into two categories: Moral Model policies and Disease Model policies.

### Moral model approach

In the early years following the revolution’s victory, Iran’s approach to substance use disorders focused on reducing people’s tendency towards substance use. Political and religious leaders consistently warned about the individual and societal harms of substance use disorders, and the use of drugs was prohibited from the perspective of Sharia. In 1980, a bill intensifying the punishment for substance-related crimes was approved, with substance consumption deemed punishable by imprisonment. That same year, the Drug Control Headquarters was established. In 1988, the Expediency Discernment Council passed a resolution titled the ‘Law on the War on Drugs,’ which classified substance use disorders as crimes. Individuals with substance use disorders were required to quit within a six-month period; otherwise, they would be sent to rehabilitation centers by judicial order for cessation. If individuals continue their substance use after quitting, they would face imprisonment, flogging, and monetary fines as outlined in Article 17 of this law. Overall, from the revolution’s onset until the end of the 1980s, this approach included the prohibition of opium cultivation, the criminalization of substance use disorders, and the enforcement of strict penal laws.

### Disease model approach

Since the early 1990s, the management of the Welfare Organization of Iran in the field of substance use disorders emphasized the need for a fundamental reevaluation of the Moral Model approach. They took action by reforming rehabilitation programs and developing the first national program for prevention, treatment, and rehabilitation of substance use disorders in 1993. In 1994, the Commission for the Study of Issues and Problems of the Moral Model highlighted the necessity of expanding prevention, health, and treatment programs for substance use disorders. It proposed the implementation of a pilot program for national prevention, treatment, and rehabilitation, along with optimal utilization of cultural facilities for raising public awareness and preventing substance use disorder tendencies. The shift towards a demand reduction approach in substance control measures gained more emphasis in the mid-1990s, accompanied by changes in theoretical foundations and practical methods. This change wasn’t sudden but took about a year and a half to take a significant and distinct form. During this period, intellectuals, writers, media, and other opinion-makers played a significant role in influencing authorities’ mindset. Failures of past campaigns and initiatives, combined with the significant impact of the HIV/AIDS epidemic in the 1990s and the readiness of policymakers and managers in the field of substance use disorders, created the groundwork for adopting new approaches and transformations among policymakers and decision-makers. In general, treatment methods for substance use disorders in Iran can be categorized into two groups: policies and treatment approaches with a voluntary and optional perspective, and policies and treatment approaches with a compulsory perspective.

### Policies and treatment approaches with a voluntary and optional perspective

These policies prioritize voluntary participation, wherein individuals with substance use disorders actively seek treatment according to their own will and desire. This approach encompasses eight types of treatment centers: outpatient substance use disorder treatment centers, inpatient substance use disorder treatment centers, medium-term residential treatment centers for substance use disorders, self-help group residential centers, long-term residential therapeutic communities (TCs), substance-assisted treatment centers for substance use disorders, behavioral disease counseling centers, and harm reduction centers (Article 15 centers). The common characteristic among all these centers is their voluntary nature.

### Policies and treatment approaches with a compulsory perspective

In these treatment approaches, individuals with substance use disorders, referred to as ‘Motejaher’ users (homeless individuals who use or deal drugs in public view [41]), are compelled to undergo treatment against their will and desire, typically through court orders and compulsory measures by law enforcement authorities (Article 16 centers).

In this section, the identified challenges in the content of treatment policies have been addressed (Table 4).

**Table 4:** Challenges of policy content

Challenges of substance use disorder treatment policies in Iran	Lack of revision in laws
	Existence of contradictions in laws and nature of disease
	Lack of evidence-based policy-making
	Absence of a comprehensive perspective on the substance use disorder phenomenon

### Lack of revision in laws

One of the challenges frequently mentioned by interviewees is the lack of revision in substance use disorder treatment laws and policies. This issue is cited as a significant factor contributing to the misuse of certain laws. Interviewees believe that aligning laws with advancements in the field of substance use disorder and with societal changes could enhance their effectiveness. However, the failure to update laws in response to current conditions negatively impacts their enforcement.

For instance, one interviewee highlighted a common problem: “One of our biggest problems is that our laws are not properly revised, or revisions are done very infrequently. This means that when our circumstances are heading towards a crisis, we suddenly remember to change the laws…” [I.3]. This reactive approach—where laws are only revised in response to crises rather than being proactively updated—exemplifies the broader issue of insufficient law revision.

### Existence of contradictions in laws and nature of disease

Another identified challenge is the existence of contradictions in laws and nature of disease. These contradictions are evident in the laws concerning substance use disorder treatment and the perspective on people with substance use disorders.

Interviewees believed that there is still a “Moral Model “approach within the framework of Disease Model. This is evident when a person with a substance use disorder is apprehended and is not a voluntary member of treatment centers; according to the law, they must be transferred to compulsory treatment centers (Article 16).

On the other hand, in laws related to harm reduction, the ultimate goal of the Disease Model approach is stated as complete abstinence. However, the philosophy of harm reduction is to reduce harm, not necessarily to achieve abstinence.

In this regard, one of the interviewees stated: “The existing laws can be interpreted in various ways; in other words, the substance control law is rife with contradictions or ambiguities that allow for interpretation. It can be exceedingly stringent, treating individuals with substance use disorders as criminals in any form. Why is it written this way? Because the authors of the law failed to align their thinking with the reality of substance use disorder as a chronic, relapsing disease. We must acknowledge relapses; we must accept that it is a chronic disease, and complete recovery from it is often unattainable.

“Yet, we even drafted harm reduction laws with an abstinence-oriented approach. We failed to grasp the philosophy behind these approaches….” [I.26].

We all know that substance use disorder is a chronic relapsing phenomenon with a high likelihood of relapse. According to Iranian laws, the ultimate goal of substance use disorder treatment is complete abstinence. However, in Article 16 (compulsory treatment), it is stated that individuals with overt substance use disorders are held in these centers for 1 to 3 months (in some cases, up to six months) and then released. They are arrested again, and this cycle continues because the time period prescribed by the law is not sufficient and contradicts the goal of treatment…” [I.14].

### Lack of evidence-based policy-making

Interviewees highlighted the lack of attention to evidence in policy decisions and the prevalence of personal, non-evidence-based opinions among individuals involved in policymaking as significant challenges in this field. This suggests that policymaking in this area is sometimes influenced more by personal opinions than by evidence. Given that policymaking is a process based on the consensus of political elites, this approach poses a challenge in this field. In this regard, one of the interviewees stated:

“The decisions we make are often influenced by our own thoughts and prior experiences in a given field. In the formulation and implementation of laws and policies, personal preferences and approaches are frequently applied instead of relying on evidence….” [I.16].

“Our policies have shortcomings; frankly, there are certain actions we should avoid. We must set aside personal biases and eliminate the phrase ‘in my opinion’ from our meetings. Instead, we should assert ‘the evidence shows that this action should be taken.’ Prior to nationwide implementation, every action should undergo a pilot phase to assess its effectiveness. If errors occur, it’s crucial to acknowledge them. Adhering to these principles can address many of our challenges…” [I.12].

### Absence of a comprehensive perspective on substance use disorder

The presence of a one-dimensional perspective is highlighted as a significant challenge in substance treatment policies by the interviewees. This suggests that policymakers view substance use disorders in a simplistic manner, lacking a comprehensive understanding. These approaches fail to account for the multifaceted nature of drug use. Consequently, decision-making and policymaking tend to be reductionist.

“Perhaps the most significant challenge in the country regarding substance use disorder is the prevalence of one-dimensional views and the failure to properly understand this phenomenon. Attempting to equate this multi-dimensional issue with sin, immorality, social misconduct, crime, behavioral disorder, or neuropsychiatric illness reflects a superficial approach. Such narrow perspectives can only explain certain aspects of the reality of this phenomenon, and any actions based on this incomplete understanding are unlikely to be effective…” [I.15].

“Each structure involved in substance use disorder treatment focuses on a specific aspect of the overall phenomenon, often overlooking many other dimensions. For instance, the Ministry of Health primarily concerns itself with harm reduction, Welfare addresses social problems, and prisons handle substance use disorder prevalence within correctional facilities. However, this arrangement presents another dilemma: none of these structures offer a comprehensive approach to substance use disorder treatment, and they lack the capacity to develop one…” [I.11].

## Discussion

Since the beginning of the Iranian Revolution in 1979, Iran has adopted a law enforcement approach to dealing with people with substance use disorders, perceiving it primarily as a crime. In other words, the prevailing perspective in Iran regarding this issue was the “Moral Model.” This stance was reinforced by the high social stigma associated with substance use disorders, prompting stricter measures in this area. In line with this policy, harsh actions and measures, such as imprisonment and execution, were implemented in the country for buying, selling, and using drugs [42]. However, after the growing population of individuals who inject drugs and the spread of the HIV/AIDS epidemic which was evidence of the ineffectiveness of previous policies and measures [43], the Iranian Welfare Organization initiated a shift towards a patient-centered approach, moving away from the earlier Moral Model strategy. While this change was supported by evidence, it may also have been influenced by individuals with differing approaches in key decision-making positions, which sometimes play a role in shaping policy shifts. These individuals argue that the punitive and tough approach to substance use disorders not only failed to rehabilitate and treat individuals but also excluded them from society, leading to increased involvement in criminal activities due to labeling and exclusion. They argue that the punitive and tough approach to substance use disorder not only failed to rehabilitate and treat individuals but also exclude them from society, leading to increased involvement in criminal activities due to labeling and exclusion [44].

Based on this, the Iranian legislature has attempted to focus on decriminalization. The laws and policies in this area define treatment as a set of evidence-based medical and non-medical interventions aimed at improving individual functioning and reintegrating the individual into society [45]. The ultimate goal of treatment is stated as achieving a life without substance use [46]. This indicates that the ultimate concept of treatment in Iranian documents and laws is interpreted as complete abstinence from any substances, whereas in many other countries, treatment and harm reduction do not necessarily require a substance-free life. Instead, the improvement of individual functioning, the reduction in the frequency of substance use, and enhanced quality of life are considered the most important criteria for treatment [18].

In line with this perspective, the Comprehensive Document on Social Support and Addiction Treatment in Iran also defines the ultimate goal of treatment as achieving a life without substance use [35]. However, this approach is influenced by the cultural context of Iran.

Iran’s cultural landscape, shaped by a blend of traditional moral values and modern perspectives, often influences attitudes toward substance use and harm reduction approaches. With its dualistic cultural background, Iranians may face tension between adopting evidence-based harm reduction strategies and adhering to more conservative, moralistic viewpoints that emphasize punitive measures. This cultural dualism can lead to resistance against harm reduction approaches, as they may be perceived as enabling or condoning substance use rather than focusing on punishment and rehabilitation. These cultural factors present significant challenges to the implementation of harm reduction policies [47–49].

Numerous studies from various countries emphasize Disease Model and treatment strategies that prioritize individual safety and well-being. These interventions aim to reduce mortality, morbidity, and harm associated with risky behaviors. As demonstrated by Zafarghandi et al., harm reduction measures were effective in decreasing high-risk behaviors in prisons [50, 51]. Such approaches acknowledge that complete abstinence may not be feasible for all individuals and instead focus on enhancing overall health while mitigating the adverse consequences of substance use [52–54].

“Kanato and colleagues also mentioned in their study that ideologically driven countries like China and Malaysia implement harm reduction and treatment interventions with the goal of complete abstinence [55]. This observation is also applicable in Iran.

Generally, substance treatment policies in Iran adhere to two main approaches, reflecting historical national-level policies. These approaches encompass compulsory treatment policies and voluntary treatment policies [56].

In compulsory approaches, individuals are placed under treatment based on a court order and the decision of a judge [41], while in voluntary approaches, individuals participate in treatment programs voluntarily. In China and Malaysia, despite evidence suggesting the ineffectiveness of compulsory treatment methods, both compulsory and voluntary approaches coexist [57–59].

The research findings reveal several challenges within the substance treatment policies of the Islamic Republic of Iran. These challenges include a lack of revision in laws, existence of contradictions in laws and nature of disease, and a deficiency in evidence-based policymaking. Additionally, our study identified a significant gap in the holistic understanding of the substance use disorder phenomenon.

Specifically, the data indicate that outdated legislation has not been adequately revised to address current challenges in substance use. For instance, participants mentioned the emergence of new drugs on the market for which there are no specific laws. The analysis shows that, according to participants’ viewpoints, many Disease Model programs are designed with a focus on drug cessation, eradication, and reduction of drug use. There is also a noted lack of evidence-based policymaking, with policy development often influenced by personal opinions and myths.

Additionally, the findings highlight further challenges, such as the influence of personal preferences and approaches in the formulation and implementation of laws and policies. There is also a notable absence of a thorough understanding of substance use disorders and a Disease Model perspective in the treatment framework. Moreover, drug use is not considered a multidimensional phenomenon in policymaking, overlooking the various factors that contribute to its occurrence. Furthermore, the study findings indicate that substance policies in low- and middle-income countries are not evidence-based. They are primarily based on the personal opinions of policymakers, often with a punitive approach rather than an evidence-based one [60, 61]. Saberi Zafarghandi et al. also point out similar issues in their study. They highlight the existence of contradictions and inconsistencies in substance use disorder policies and laws, as well as the lack of evidence-based decision-making. Policymaking in this field is often driven by the personal opinions of policymakers [62].

### Limitations

This study has several limitations that should be acknowledged. First, the analysis was limited to publicly accessible policy documents, which may not include all relevant policies, especially informal or unpublished ones. Second, the reliance on interviews with policymakers and implementers introduces the potential for bias, as the perspectives of other key actors, such as healthcare providers or individuals affected by substance use disorder, were not explored. Third, this study’s findings are rooted in the cultural, social, and political context of Iran, which may limit the generalizability of the results to other countries with different substance use frameworks. Lastly, qualitative content analysis, while rigorous, is inherently subjective, and alternative interpretations of the data could lead to different conclusions.

## Conclusion

In general, the policy landscape for substance use disorder treatment in Iran has evolved through two periods: the “Moral Model” and “Disease Model”. However, delineating these periods from each other is not straightforward. The field of substance use disorder policy in Iran faces multiple challenges. The presence of diverse stakeholders with varying definitions and perspectives on substance use disorder and its treatment has hindered the formation of a unified and progressive discourse in policymaking.

Moreover, the influence of personal preferences in policymaking and the lack of evidence-based approaches have contributed to weaknesses in decision-making and the formulation of appropriate policies. Substance use disorder treatment policies in Iran contain contradictions and ambiguities that allow for interpretation by implementers. Despite negative experiences with the “Moral Model,” inappropriate patterns such as compulsory treatments (Article 16 centers) still persist.

It seems that the true philosophy of Disease Model has not been fully comprehended by health policymakers in Iran. All Disease Model policies are pursued with a focus on abstinence and quitting, possibly stemming from the stigma associated with this issue.

Iran, like other countries grappling with similar challenges, needs to rely more on evidence-based approaches and shift away from the “Moral Model” paradigm to adopt more effective substance use disorder treatment policies.

## Data Availability

The data that support the findings of this study are available from the corresponding author upon reasonable request.
